# Resolving indexing ambiguities in X-ray free-electron laser diffraction patterns

**DOI:** 10.1107/S2059798318013177

**Published:** 2019-02-06

**Authors:** Monarin Uervirojnangkoorn, Artem Y. Lyubimov, Qiangjun Zhou, William I. Weis, Axel T. Brunger

**Affiliations:** a Linac Coherent Light Source, SLAC National Accelerator Laboratory, Menlo Park, CA 94025, USA; b Stanford Synchrotron Radiation Laboratory, SLAC National Accelerator Laboratory, Menlo Park, CA 94025, USA; c Department of Molecular and Cellular Physiology, Stanford University, Stanford, CA 94305, USA; d Department of Neurology and Neurological Science, Stanford University, Stanford, CA 94305, USA; e Department of Structural Biology, Stanford University, Stanford, CA 94305, USA; f Department of Photon Science, Stanford University, Stanford, CA 94305, USA; g Howard Hughes Medical Institute, Stanford University, Stanford, CA 94305, USA

**Keywords:** serial crystallography, indexing ambiguity, XFELs

## Abstract

Processing X-ray free-electron laser (XFEL) diffraction images poses challenges, as an XFEL pulse is powerful enough to destroy or damage the diffracting volume and thereby yields only one diffraction image per volume. Moreover, the crystal is stationary during the femtosecond pulse, so reflections are generally only partially recorded. Therefore, each XFEL diffraction image must be scaled individually and, ideally, corrected for partiality prior to merging. An additional complication may arise owing to indexing ambiguities when the symmetry of the Bravais lattice is higher than that of the space group, or when the unit-cell dimensions are similar to each other. Here, an automated method is presented that diagnoses these indexing ambiguities based on the Brehm–Diederichs algorithm [Brehm & Diederichs (2014 ▸), *Acta Cryst.* D**70**, 101–109] and produces a consistent indexing choice for the large majority of diffraction images. This method was applied to an XFEL diffraction data set measured from crystals of the neuronal SNARE–complexin-1–synaptotagmin-1 complex. After correcting the indexing ambiguities, substantial improvements were observed in the merging statistics and the atomic model refinement *R* values. This method should be a useful addition to the arsenal of tools for the processing of XFEL diffraction data sets.

## Abstract 2

An automated method is presented that diagnoses indexing ambiguities and produces a consistent indexing choice for the large majority of diffraction images.

## 1. Introduction   

An XFEL pulse generated by the Linac Coherent Light Source (LCLS) typically delivers ∼10^11^ photons in ∼40 fs. These ultrafast and ultrabright pulses produce diffraction patterns of single small crystals or diffraction volumes within larger crystals, but at the cost of damaging the diffracted crystal or volume. This situation is akin to the ‘one crystal–one photograph’ condition, a term that was first introduced by Winkler *et al.* (1979 ▸) in their work on processing multi-crystal diffraction data from tomato bushy stunt virus. In that work (and independently by Rossmann *et al.*, 1979 ▸), the partialities of the observed intensities were estimated and used to correct the intensities to their fully recorded equivalents by post-refinement of the parameters that determine partiality. The rotation technique used in these pioneering studies enabled the recording of the full intensities of a subset of Bragg reflections, which could be used as a reference set for parameter refinement. In contrast, the crystal is stationary while exposed to the femtosecond XFEL pulse and only one such still image is obtained per crystal, so data collection produces two-dimensional slices of Bragg reflections at random crystal orientations. For typical macromolecular crystal mosaicities and X-ray energy bandpasses, all recorded XFEL reflection intensities are partial. Moreover, each of these still images has to be individually indexed in order to obtain the crystal orientation and unit-cell dimensions, and then individually scaled, integrated, corrected for partiality and merged in order to obtain a complete diffraction data set. By collecting many thousands to millions of diffraction images, the so-called Monte Carlo method (Kirian *et al.*, 2010 ▸) reduces the need for precise scaling and post-refinement. Nevertheless, if the crystal quantities and/or XFEL beamtime are limited, post-refinement is essential to obtain the best possible diffraction data (Uervirojnangkoorn *et al.*, 2015 ▸). Several post-refinement methods for XFEL diffraction data have been developed (White, 2014 ▸; Kabsch, 2014 ▸; Uervirojnangkoorn *et al.*, 2015 ▸; Ginn *et al.*, 2015 ▸; Kroon-Batenburg *et al.*, 2015 ▸) and successfully applied to several XFEL diffraction data sets.

Prior to post-refinement and merging, individual diffraction images must be indexed consistently with respect to one another. When there are inconsistencies in indexing choices among images, the statistical properties of the merged diffraction data are abnormal. For example, diffraction data sets with mixed indexing choices may appear to be twinned after merging and produce high model *R* values and poor-quality electron-density maps (Brehm & Diederichs, 2014 ▸). For continuous-rotation data measured from a single crystal, an indexing choice is set at the beginning of the rotation series and all images are consistently indexed. In contrast, for rotation or still data obtained from multiple crystals, each data set or diffraction image is indexed independently, which can result in inconsistent indexing choices. Methods have been developed to resolve the indexing ambiguity for merohedral space groups that have a lower symmetry than the Bravais lattice using pairwise similarities (Brehm & Diederichs, 2014 ▸) or expectation-maximization algorithms (Liu & Spence, 2014 ▸). These methods have been successfully used to resolve indexing ambiguities for several XFEL diffraction data sets (Spence, 2017 ▸).

Non-merohedral space groups may also be prone to ambiguities in some cases. For example, a pseudo-tetragonal crystal setting, such as an orthorhombic crystal with a pair of axes with similar lengths, can yield two nearly identical indexing solutions. These indexing ambiguities can be resolved with existing methods (Brehm & Diederichs, 2014 ▸). We have developed an automated procedure that recognizes both merohedral and pseudo-merohedral indexing ambiguities and corrects them using the method of Brehm & Diederichs (2014 ▸). Moreover, we implemented a bootstrap procedure that uses only a subset of the diffraction images to calculate the correlation matrix used in the Brehm–Diederichs algorithm.

We applied this method to process XFEL diffraction images obtained from crystals of the neuronal SNARE–complexin-1–synaptotagmin-1 complex (Zhou *et al.*, 2017 ▸) in order to resolve the indexing ambiguity that arose from two similar unit-cell dimensions in space group *P*2_1_2_1_2. Using a subset of 100 images yielded an indexing-ambiguity solution that was almost identical to the solution obtained from all 324 images. This result illustrates the efficiency of our method, which can be used even in the case of large XFEL diffraction data sets obtained using liquid jet sample delivery techniques.

## 2. Methods   

### 2.1. XFEL diffraction data collection   

The SNARE–complexin-1–synaptotagmin-1 complex crystals were grown as described previously (Zhou *et al.*, 2017 ▸). The XFEL diffraction data were collected at the Macromolecular Femtosecond Crystallography (MFX) endstation of the LCLS at the SLAC National Accelerator Laboratory using a goniometer-based target sample-delivery station and an automatic sample-loading system designed and adapted for XFEL diffraction experiments (Cohen *et al.*, 2014 ▸). We used a 30 µm XFEL beam with a pulse duration of 40 fs in the self-amplified stimulated emission (SASE) mode (Kondratenko & Saldin, 1979 ▸; Bonifacio *et al.*, 1984 ▸). The energy spectrum for each shot was recorded and the centroid of the SASE energy spectrum was used as the wavelength input to the indexing and integration and post-refinement steps. A total of 80 crystals were screened, yielding 400 images with usable diffraction.

### 2.2. Indexing and integration of the XFEL diffraction images   

The diffraction images were indexed and integrated using *cctbx.xfel* (Hattne *et al.*, 2014 ▸) with optimization algorithms as implemented in *IOTA* v.1.1.013 (Lyubimov, Uervirojnang­koorn, Zeldin, Brewster *et al.*, 2016 ▸). A beamstop shadow mask for the diffraction patterns was created by identifying pixels with intensities below 5 ADU (analog-to-digital units; this value was determined by inspecting the lowest background values of a few good images). For the spot-finding grid search the spot area was set to 12 ± 1 pixels and the spot signal height was set to 7 ± 1 (a total of nine integration attempts per diffraction image). In the initial runthrough, the indexing and integration steps were carried out without using any *a priori* unit-cell or crystal-symmetry information. This yielded a total of 338 integrated images; the majority of these (195 images) were indexed in the tetragonal Bravais lattice *P*4 and 70 images were indexed in an orthorhombic Bravais lattice (*P*222).

While we were processing the XFEL diffraction data, we also determined the crystal structure of the SNARE–complexin-1–synaptotagmin-1 complex to 1.85 Å resolution using diffraction data collected on a synchrotron microfocus beamline (Zhou *et al.*, 2017 ▸; see below). The space group for this synchrotron data set is *P*2_1_2_1_2 and the unit-cell dimensions are *a* = 85.7, *b* = 89.7, *c* = 91.7 Å (Table 1 ▸). We subsequently used the unit-cell dimensions of the synchrotron data set as a target unit cell for a second trial of indexing and integration of the XFEL data set; the unit-cell and symmetry information were also used to direct the integration-optimization procedure in *IOTA*. This second trial of indexing and integration yielded 324 integrated images in the orthorhombic Bravais lattice *P*222 with similar unit-cell dimensions to those of the synchrotron data.

### 2.3. Resolving the indexing ambiguity of the XFEL diffraction data   

After integration, the XFEL diffraction data were scaled, post-refined and merged. The post-refinement parameters are provided in Table 1 ▸. From the *IOTA* integration statistics, 316 integrated images had measurable diffraction to 2.0 Å resolution. We used this as the limiting resolution and applied an *I*/σ(*I*) > −3 cutoff for post-refinement and merging using the merging program *PRIME* (Uervirojnangkoorn *et al.*, 2015 ▸). The initial scaling was performed on each image using the pseudo-Wilson scaling method (Lyubimov, Uervirojnang­koorn, Zeldin, Zhou *et al.*, 2016 ▸); only reflections with *I*/σ(*I*) > 2 and in the resolution (*d*_min_) range 2.5–5.0 Å were used to determine the initial scale factor (*G*_0_) and temperature factor (*B*_0_).

**Figure 1:**
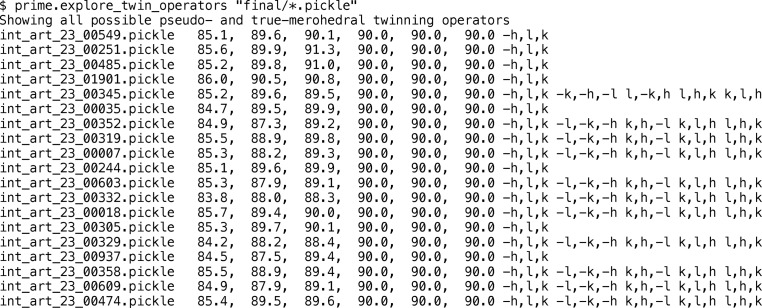
Possible re-indexing operators for a given unit cell per diffraction image. A representative sample is shown for the 324 diffraction images of the SNARE–complexin-1–synaptotagmin-1 XFEL diffraction data set. The *prime.explore_twin_operators* program was used to determine the possible indexing operators starting from the integrated intensities using *IOTA* (Lyubimov, Uervirojnangkoorn, Zeldin, Zhou *et al.*, 2016 ▸).

We developed a program, *prime.explore_twin_operators*, to identify indexing ambiguities in the diffraction data set. The program tests for potential merohedral or pseudo-merohedral ambiguities, with the latter being relevant for this diffraction data set (space group *P*2_1_2_1_2). Only the unit-cell dimensions are needed to run the program, and a representative output is shown in Fig. 1 ▸. The program indicated that 317 of the 324 diffraction images could be re-indexed with the operator −*h*, *l*, *k*. Additional indexing choices (−*l*, −*k*, −*h*; *k*, *h*, −*l*; *k*, *l*, *h*; *l*, *h*, *k*) were indicated for a small subset of the diffraction images, suggesting that these diffraction images could be re-indexed with cubic lattice symmetry. We note that the number of indexing choices may vary when changing the Le Page delta parameter (Le Page, 1982 ▸), since this parameter determines whether the unit-cell dimensions are compatible with a particular space group. We set this parameter (using the --max_delta option available in *prime.explore_twin_operators*) to a fairly generous 3° to account for the inherent variability of the unit-cell dimensions estimated from serial diffraction images. However, the large differences between the *a* versus the *b* and *c* unit-cell dimensions make it unlikely that this small subset of images was derived from cubic crystals and that these additional operators represent true alternative indexing choices for this small subset of diffraction images. Therefore, all diffraction images were tested for re-indexing with the indexing operator −*h*, *l*, *k*. Methods for identifying these possible operators have been described elsewhere (Zwart *et al.*, 2008 ▸).

Given their similar values, the unit cell *b* and *c* dimensions were arbitrarily assigned during the indexing step for each still diffraction image. In general, if the unit cell dimensions are very different one can use the Niggli reduced cell to swap the *b* and *c* axes such that all diffraction images have the same indexing choice. However, for this particular data set the *b* and *c* axis dimensions were very similar. Owing to the variations in the initial indexing parameters, one of the two dimensions may appear to be smaller or larger than its true value. These variations in the unit-cell dimensions made it impossible to use the Niggli reduced cell settings for merging. Thus, we used the Brehm–Diederichs algorithm to identify the correct indexing choice.

In the Brehm–Diederichs algorithm, a vector of reflections in one diffraction image is projected into a *k*-dimensional space as a point, where *k* corresponds to the number of possible indexing choices (*k* = 2 for the particular data set used here). Initially, the coordinate of each point is assigned randomly. A target function is defined so that these points gradually move towards a cluster in which the diffraction intensities are more correlated (and, conversely, as far away as possible from the cluster in which they are less correlated). Brehm and Diederichs provide two target functions: (i) minimization of the length and (ii) minimization of the scalar product (the length and the angle) between these points. We chose to use the latter (defined by equation 3 in Brehm & Diederichs, 2014 ▸),
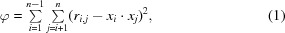
where *n* is number of diffraction images, *r*_*i*,*j*_ is the Pearson correlation between diffraction images *i* and *j*, and *x_i_* is the coordinate of diffraction image *i*.

**Figure 2:**
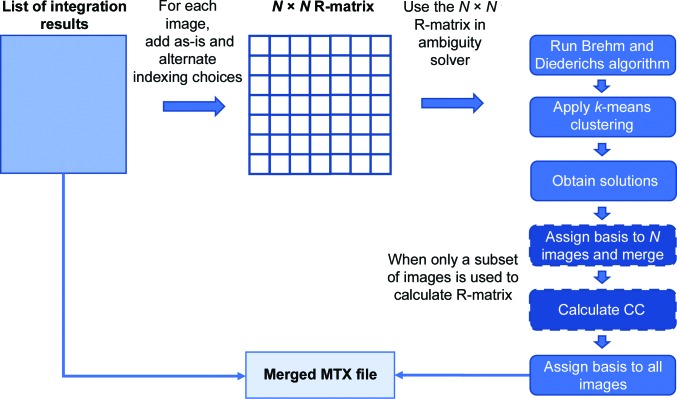
Flowchart of the indexing-ambiguity-resolving algorithm. Once (an) alternative indexing choice(s) is/are selected, the program calculates the target function (1). A cluster is selected using the *k*-means algorithm and the solutions are passed on to the merging step. The two steps with dashed outlines indicate additional steps if a subset of the diffraction images is used for the resolution of the indexing ambiguities, instead of the full diffraction data set. In this case, this subset is merged to form a reference data set that is used to bootstrap the determination of the indexing operator for the remaining diffraction images (see Section 2.3).

**Figure 3:**
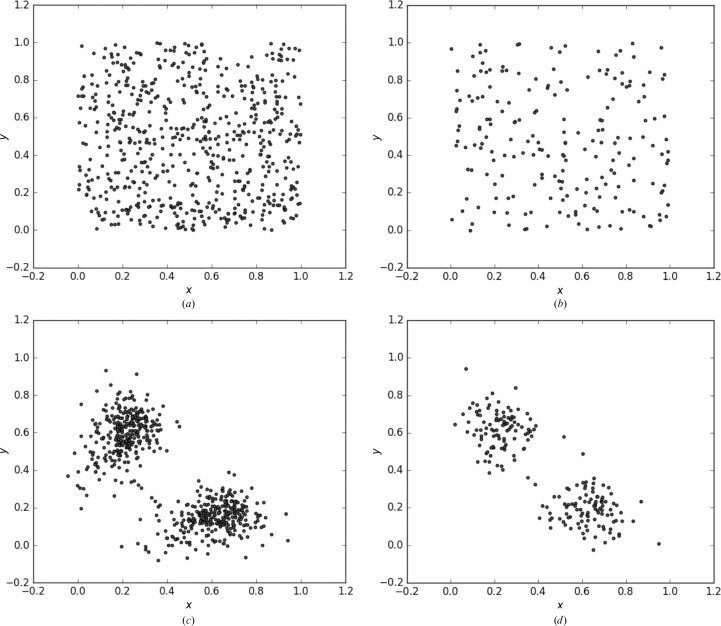
Results of the index ambiguity resolving algorithm as implemented by Brehm & Diederichs (2014 ▸). (*a*) Random coordinates chosen initially for the 324 integration results with both *h*, *k*, *l* and −*h*, *l*, *k* indexing choices (648 points in total). (*b*) Initial random coordinates for a subset of 100 images (200 points in total). Two distinct clusters result from the minimization algorithm when all images were used (*c*) and when only a subset of 100 images was used (*d*).

Once the indexing choices had been determined, we proceeded with the indexing ambiguity resolving process (Fig. 2 ▸) by calculating the residual matrix (1) for both indexing choices (*h*, *k*, *l* and −*h*, *l*, *k*) on the partiality-corrected intensities and included both indexing choices in a superset of the diffraction data. The result revealed two clusters of diffraction images with the indexing choice that makes their intensities correlate best with the population in the same cluster (Figs. 3 ▸ *c* and 3 ▸ *d*). The algorithm identified the centroids of these clusters. The diffraction images that belong to each of the two clusters were then grouped together and one of the two groups was arbitrarily selected for merging.

We also tested using a subset of randomly chosen diffraction images to resolve the indexing ambiguities and then resolving the ambiguities of the remaining data by a bootstrap procedure. The number of diffraction images used in this subset depends on different factors such as the number of reflections that were detectable in the diffraction patterns, the unit cell dimensions and the space group. For this particular data set, we found that a subset of 100 diffraction images was sufficient for the bootstrap procedure. In this approach, the subset is integrated, the indexing ambiguities are resolved and the resulting data are post-refined. This merged data set is then used to calculate the Pearson correlation coefficient between each of the remaining integrated images indexed in either of the indexing choices (*h*, *k*, *l* and −*h*, *l*, *k* for the SNARE–complexin-1–synapto­tagmin-1 data set) and the current merged data set. The indexing operator of the diffraction data set that produces the higher value of the Pearson correlation coefficient is selected as the correct indexing choice. To obtain the final merged diffraction data set, all images with their obtained indexing solution are post-refined and merged together. Fig. 3 ▸ shows the starting points and the results after the minimization of (1) when all images were used (Figs. 3 ▸ *a* and 3 ▸ *c*) and when only 100 images were used (Figs. 3 ▸ *b* and 3 ▸ *d*). We observed two distinct clusters in each case. In the case of the 100 image subset, we post-refined and merged the selected images to obtain a reference data set, which was then used to determine the indexing choice for the rest of the XFEL diffraction images.

**Figure 4:**
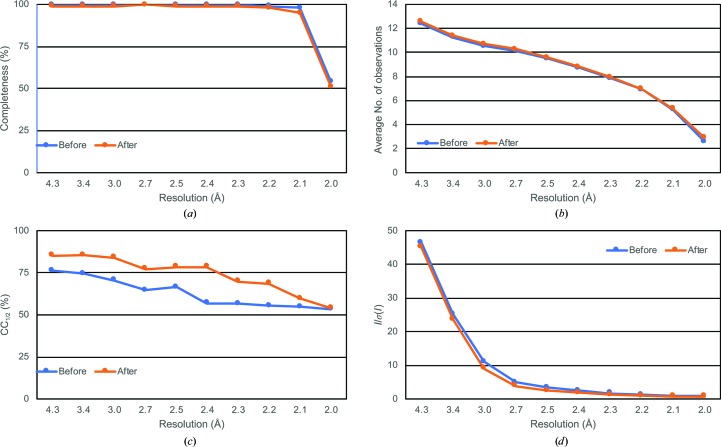
Merging statistics obtained from post-refinement and merging of diffraction patterns before and after the index-ambiguity-resolving procedure. (*a*) Completeness. (*b*) Average number of observations. (*c*) CC_1/2_. (*d*) *I*/σ(*I*) of the merged diffraction data.

**Figure 5:**
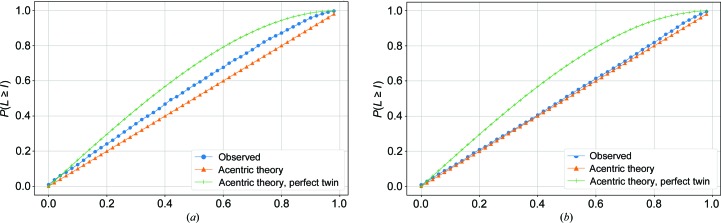
|*L*|-test plots for an XFEL diffraction data set of the SNARE–complexin-1–synaptotagmin-1 complex. (*a*) Merged diffraction data set processed without resolving the pseudo-merohedral indexing ambiguity of this data set. (*b*) Merged reflection data set processed after resolving the indexing ambiguity.

After applying the newly determined re-indexing operators and performing post-refinement and merging, the completeness, average number of observations and *I*/σ(*I*) remained very similar (Figs. 4 ▸ *a*, 4 ▸ *b* and 4 ▸ *d*, Table 1 ▸). This is as expected since these metrics are primarily determined by the number of observed reflections, independent of indexing choices. However, resolving the indexing ambiguity resulted in increased values of CC_1/2_ across all resolution bins. Correspondingly, the overall CC_1/2_ increased by ∼9.6%, from 79.1% to 87.7%. The *L*-test of the re-indexed data set also showed no abnormal behavior (Fig. 5 ▸).

### 2.4. Synchrotron data collection and processing   

The synchrotron diffraction data collection for the SNARE–complexin-1–synaptotagmin-1 complex has been described previously (Zhou *et al.*, 2017 ▸) and is briefly summarized here. The synchrotron data were collected on beamline 24-ID-C of the Advanced Photon Source (APS) at Argonne National Laboratory, Argonne, Illinois, USA. A 70 µm beam (3 × 10^12^ photons s^−1^ was used throughout the experiment with a rotation of 0.2° per frame and an exposure time of 0.2 s. Diffraction data from the best crystals were indexed and integrated using *XDS* (Kabsch, 2010 ▸) and were scaled and merged using *SCALA* (Evans, 2006 ▸) (Table 1 ▸).

## 3. Results and discussion   

The synchrotron diffraction data from crystals of the SNARE–complexin-1–synaptotagmin-1 complex revealed that the crystals were orthorhombic (Table 1 ▸; Zhou *et al.*, 2017 ▸). Using this information to process the XFEL data set resulted in a total of 324 integrated XFEL diffraction images. Scaling, post-refinement and merging yielded a merged data set with an overall completeness of 93.7% at a resolution of 2.0 Å (and a completeness of 42.9% for the 2.07–2.0 Å resolution bin), an overall CC_1/2_ of 79.1% and a mean |*L*| of 0.443 (Table 1 ▸). The integration results were merged with the inclusion of negative measurements in order to prevent abnormal behavior of the *L*-test result caused by data truncation (Lyubimov, Uervirojnangkoorn, Zeldin, Zhou *et al.*, 2016 ▸); with this in mind, it was alarming to find that the mean |*L*| value was abnormal (Fig. 5 ▸ *a*). Moreover, the high *R* values from atomic model refinement (*R*_work_ and *R*_free_ of 41.3% and 44.4%, respectively) suggested a problem with the XFEL data set.

The *b* and *c* axes of the unit cell of the SNARE–complexin-1–synaptotagmin-1 complex crystal are very similar to each other (Table 1 ▸): the initial indexing and integration statistics reported by *IOTA* revealed mean values and standard deviations of *b* = 88.8 ± 1.0 Å and *c* = 89.7 ± 0.7 Å. The small differences in these mean values and the relatively large standard deviations suggested that the merged data set contained mixtures of different indexing choices. To resolve this ambiguity, we generalized the Brehm–Diederichs algorithm (Brehm & Diederichs, 2014 ▸) in *PRIME* (Uervirojnangkoorn *et al.*, 2015 ▸) to include this type of pseudo-merohedral ambiguity (Section 2.3). This corrected diffraction data set produced an overall CC_1/2_ of 86.8% and a mean |*L*| of 0.488 (Table 1 ▸; Fig. 5 ▸ *a*), which represented a substantial improvement over the initial data-processing results. We determined the structure by molecular replacement using the structure of the SNARE–complexin-1–synaptotagmin-1 complex obtained from synchrotron diffraction data as the search model (Table 1 ▸; PDB entry 5w5c; Zhou *et al.*, 2017 ▸). Several cycles of model building and refinement yielded an *R*_work_ and *R*_free_ of 28.9% and 29.6%, respectively, which were much improved compared with the data set prior to resolving the indexing ambiguities (Table 1 ▸, column 1).

**Figure 6:**
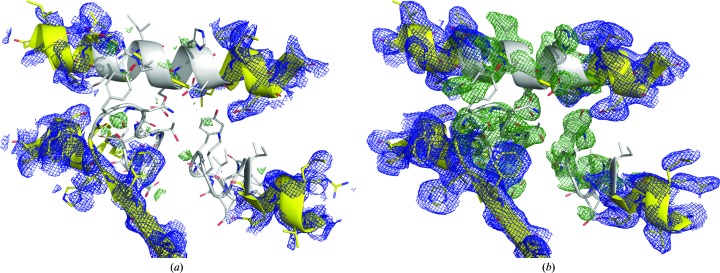
2*mF*_o_ − *DF*_c_ electron-density maps (in blue; contoured at 1.6σ) and the simulated-annealing composite (*mF*_o_ − *DF*_c_) OMIT maps (in green; contoured at 3.0σ) using the XFEL diffraction data for the SNARE–complexin-1–synaptotagmin-1 complex. These OMIT maps were generated by setting the occupancies of 30 residues (shown in white) in three different chains (chain *B*, residues 206–215; chain *E*, residues 65–74; chain *F*, residues 379–388) to zero prior to atomic model refinement. These maps were generated from (*a*) the merged diffraction data set processed without resolving the indexing ambiguity and (*b*) the merged diffraction data set processed with indexing-ambiguity solutions.

The impact of resolving the indexing ambiguity can also be seen in simulated-annealing composite (*mF*_o_ − *DF*_c_) OMIT maps (Fig. 6 ▸; in green; contoured at 3.0σ). The *mF*_o_ − *DF*_c_ OMIT map generated from the diffraction data set with the corrected indexing choices (Fig. 6 ▸ *b*) shows strong, well resolved positive electron density that covers most of the omitted residues. On the other hand, the merged diffraction data set with initial mixed indexing choices shows essentially no positive difference density around the omitted region (Fig. 6 ▸ *a*).

Although the XFEL diffraction data set was much improved after resolving the indexing ambiguity, the *R* values of the model refined against these data still compare un­favorably with the same structure derived from rotation data obtained using synchrotron radiation, which resulted in an *R*_work_ and *R*_free_ of 19.4% and 23.1%, respectively (Table 1 ▸). The origin of these somewhat poorer *R* values could arise in part from errors in diffraction parameters that could not be corrected entirely during the post-refinement process. In addition, since the XFEL diffraction data set is merged from the diffraction patterns obtained from 324 crystals, structural heterogeneity could negatively affect both the merging statistics for the complete data set and the refinement residuals for the finished structure.

When using a subset of 100 XFEL diffraction images to resolve the indexing ambiguity, the merging statistics were nearly identical to those when all images were used to solve the ambiguity. The correlation coefficient of the intensities from the two data sets is 99.8%. When refining the atomic model against this merged reflection set, we obtained an *R*_work_ and *R*_free_ of 28.9% and 29.7%, respectively, which are similar to the values obtained when all images were used to resolve the indexing ambiguity. This suggests that using only a subset of images in the Brehm–Diederichs algorithm, followed by the bootstrap procedure described in Section [Sec sec2]2, was sufficient to obtain a solution for the indexing-ambiguity problem.

In summary, we have generalized the method of Brehm & Diederichs (2014 ▸) to include pseudomerohedral indexing ambiguities in an automated fashion, and implemented it as an additional tool in the post-refinement program *PRIME*, which is part of the current *PHENIX* software suite (Adams *et al.*, 2010 ▸). For the XFEL diffraction data from the SNARE–complexin-1–synaptotagmin-1 complex, our method resulted in major improvements to the merging statistics, *R* values after atomic model refinement and OMIT electron-density maps.

## Figures and Tables

**Table 1:** Data statistics Values in parentheses are for the outer shell.

	XFEL data		
	Before resolving indexing ambiguities	After resolving indexing ambiguities	Synchrotron data
Source	LCLS, SLAC	LCLS, SLAC	APS
No. of images	324	324	
Space group	*P*2_1_2_1_2	*P*2_1_2_1_2	*P*2_1_2_1_2
*a*, *b*, *c* (Å)	85.3, 88.8, 89.9	85.2, 88.8, 89.7	85.7, 89.7, 91.7
Resolution (Å)	45.0–2.00 (2.07–2.00)	45.0–2.00 (2.07–2.00)	62.6–1.85 (1.92–1.85)
Data cutoff [*I*/σ(*I*)]	−3	−3	−3
Completeness (%)	93.7 (42.9)	92.6 (42.3)	99.8 (97.2)
Multiplicity (rotation)	—	—	17.8 (18.4)
Multiplicity (still)	7.9 (2.1)	8.4 (2.4)	—
Mean |*L*|	0.443	0.488	
Post-refinement parameters			
Linear scale factor *G*_0_	2.8	3.3	—
*B* (Å^2^)	34.7	20.9	—
γ_0_ (Å^−1^)	0.0001	0.0001	—
γ_e_ (Å^−1^)	0.0029	0.0030	—
Average *T*_pr_	1751.2	1565.9	—
Average *T*_*xy*_ (mm^2^)	3.6	5.2	—
CC_1/2_ (%)	79.1 (62.3)	88.7 (62.2)	—
*R*_merge_ (rotation) (%)	—	—	9.6 (77.0)
*R*_merge_ (still) (%)	77.0 (78.2)	68.3 (78.1)	—
*I*/σ(*I*)	10.1 (1.6)	10.3 (1.3)	20.6 (0.9)
Structure-refinement parameters			
*R*_merge_/*R*_work_ (%)	41.3/44.4	28.9/29.6	19.4/23.1
R.m.s. deviations			
Bond lengths (Å)	0.010	0.008	0.016
Bond angles (°)	1.117	0.859	1.55
No. of atoms			
Protein	3908	3902	3756
Mg^2+^	1	1	1
*B* factors (Å^2^)			
Protein	57.7	77.2	77.7
Mg^2+^	40.3	66.4	69.4
